# A Novel Approach To Identify Inhibitors of Iron Acquisition Systems of Pseudomonas aeruginosa

**DOI:** 10.1128/spectrum.02437-22

**Published:** 2022-09-13

**Authors:** Mamie Kannon, N. Miranda Nebane, Pedro Ruiz, Sara McKellip, Paige N. Vinson, Avishek Mitra

**Affiliations:** a Department of Microbiology and Molecular Genetics, Oklahoma State University, Stillwater, Oklahoma, USA; b High Throughput Screening Center, Southern Research, Birmingham, Alabama, USA; University of North Carolina at Chapel Hill

**Keywords:** *Pseudomonas aeruginosa*, iron acquisition inhibitor, heme, ferrous, whole cell, high-throughput screen, drug discovery, heme transport, high throughput, inhibitor, iron acquisition

## Abstract

Pseudomonas aeruginosa is an opportunistic pathogen that has been declared by the World Health Organization as a “priority 1 critical pathogen” needing immediate new strategies for chemotherapy. During infection, P. aeruginosa uses redundant mechanisms to acquire ferric, heme (Hm), or ferrous iron from the host to survive and colonize. Significant efforts have been undertaken to develop siderophore blockers to inhibit ferric iron acquisition by P. aeruginosa, but there is a lack of inhibitors that can block Hm or ferrous iron acquisition by P. aeruginosa. We developed and employed a targeted high-throughput screen (HTS) and identified a molecule(s) that can specifically inhibit the Hm and ferrous iron acquisition systems of P. aeruginosa. Our targeted approach relies on screening a small-molecule library against P. aeruginosa under three growth conditions, where the only variable was the iron source (ferric, Hm, or ferrous iron). Each condition served as a counterscreen for the other, and we identified molecules that inhibit the growth of P. aeruginosa in the presence of only Hm or ferrous iron. Our data indicate that econazole, bithionate, and raloxifene inhibit the growth of P. aeruginosa in the presence of Hm and that oxyquinoline inhibits the growth of P. aeruginosa in the presence of ferrous iron. These iron-specific inhibitors do not interfere with the activity of meropenem, a commercial antipseudomonal, and can also increase meropenem activity. In conclusion, we present a proof of concept of a successful targeted conditional screening method by which we can identify specific iron acquisition inhibitors. This approach is highly adaptable and can easily be extended to any other pathogen.

**IMPORTANCE** Since acquiring iron is paramount to P. aeruginosa’s survival and colonization in the human host, developing novel strategies to block the access of P. aeruginosa to host iron will allow us to starve it of an essential nutrient. P. aeruginosa uses siderophore, heme, or ferrous iron uptake systems to acquire iron in the human host. We have developed a novel approach through which we can directly identify molecules that can prevent P. aeruginosa from utilizing heme or ferrous iron. This approach overcomes the need for the *in silico* design of molecules and identifies structurally diverse biologically active inhibitor molecules. This screening approach is adaptable and can be extended to any pathogen. Since Gram-negative pathogens share many similarities in iron acquisition at both the mechanistic and molecular levels, our screening approach presents a significant opportunity to develop novel broad-spectrum iron acquisition inhibitors of Gram-negative pathogens.

## INTRODUCTION

Pseudomonas aeruginosa is a ubiquitous Gram-negative opportunistic pathogen that resides in a wide range of environments (1). It is the leading cause of nosocomial infections and ventilator-associated pneumonia in the United States and is associated with extremely high mortality rates ranging from 13 to 50% (2–4). P. aeruginosa also causes chronic pulmonary infections in the lungs of patients with cystic fibrosis (CF) and results in the death of 80% of CF patients (5, 6). Recent CDC estimates show that yearly, there are ~51,000 hospital-associated P. aeruginosa infections, with 13% of these infections being caused by multidrug-resistant (MDR) P. aeruginosa. The long treatment times for MDR and pandrug-resistant (PDR) P. aeruginosa increase the risk of selecting for new resistant strains and pose a large economic burden, with average patient treatment costs being as high as $67,000 (7). Due to the numerous mechanisms of resistance of P. aeruginosa to antibiotics, a serious roadblock to treatment has been the lack of drugs inhibiting the growth of or killing P. aeruginosa by new molecular mechanisms. The combination of ceftolozane-tazobactam (C/T) was the last FDA-approved treatment for drug-resistant P. aeruginosa in 2014, and C/T-resistant P. aeruginosa was identified the same year (8). Mechanisms of resistance to all known antibiotic agents have been identified in P. aeruginosa, leading the World Health Organization to declare P. aeruginosa as a “priority 1 critical pathogen” needing new strategies and options for prevention and chemotherapy (9).

An attractive area of research to develop novel antipseudomonals has been to inhibit iron acquisition by P. aeruginosa. Iron is an essential micronutrient for most living organisms, and P. aeruginosa is completely dependent on iron acquisition to successfully colonize the human host (10–12). To restrict the growth of any invading pathogens, the human host sequesters iron within heme (Hm) or in high-affinity binding proteins such as transferrin (Tf), lactoferrin (Lf), or ferritin (13, 14). P. aeruginosa overcomes iron limitation in the host by (i) secreting the siderophores pyoverdine and pyochelin (12) to chelate ferric iron from host Tf, Lf, or ferritin; (ii) secreting a hemophore to capture Hm iron from host hemoproteins (12); or (iii) scavenging ferrous iron (15). Since it is known that pyochelin (16) and pyoverdine (10, 17–20) are required for efficient colonization by P. aeruginosa in murine infection models, considerable efforts have been dedicated to the identification of molecules that inhibit siderophore-mediated iron acquisition (SMIA) of P. aeruginosa (21). However, in clinical trials, these siderophore inhibitors are only partially effective in restricting P. aeruginosa infections (21) because P. aeruginosa still utilizes the Hm and ferrous iron uptake systems to acquire iron from the host. Notably, Hm is the largest (22) source of host iron, and the CF lung contains large amounts of ferrous iron (39 μM) (23). Moreover, previous studies have clearly shown that P. aeruginosa siderophore mutants successfully colonize the lungs of CF patients (11, 24, 25) and that P. aeruginosa downregulates siderophore production and upregulates Hm and ferrous iron uptake genes in the CF lung (11, 23). These observations collectively show that P. aeruginosa uses redundant iron uptake pathways *in vivo*. Therefore, to make the inhibition of iron acquisition a viable strategy for antipseudomonal chemotherapy, all three (siderophore, Hm, and ferrous iron) mechanisms of iron acquisition must be inhibited simultaneously *in vivo*. The fact that inhibitors of P. aeruginosa Hm and ferrous iron uptake pathways have not been developed represents a major gap in our ability to inhibit iron acquisition by P. aeruginosa, and the goal of our study was to address this knowledge gap. In this study, we developed a conditional screening approach to identify inhibitors of P. aeruginosa Hm or ferrous iron utilization systems. We present a proof of concept that this conditional screening approach can be employed to identify molecules that can specifically inhibit Hm or ferrous iron utilization by P. aeruginosa.

## RESULTS

### Growth conditions for developing the high-throughput screening assay.

**FIG 1:**
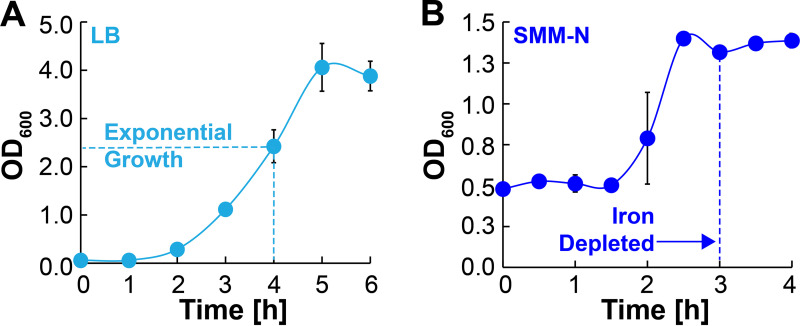
Iron depletion in P. aeruginosa. (A) Growth of wild-type P. aeruginosa PAO1 in LB broth to determine exponential and stationary growth phases of PAO1. (B) To deplete internal iron reserves of PAO1, mid-log-phase cells in LB broth were inoculated into succinate MOPS medium (SMM) containing no iron (SMM-N) and allowed to grow until there was no change in the optical density. Error bars represent the standard errors of the means (SEM) from three biological replicates. In many cases, the error bars are smaller than the marker data points.

The main goal of this study was to identify inhibitors of Hm and ferrous iron acquisition by P. aeruginosa. To achieve this goal, we developed a targeted whole-cell high-throughput screening (HTS) assay. In this HTS approach, a compound library is simultaneously screened for antipseudomonal activity under three separate growth conditions, where the only variable is the iron source (ferric chloride [FeCl_3_], hemin [Hm], or ferrous sulfate [FeSO_4_]) in the growth medium. Before the inoculation of P. aeruginosa into the HTS medium, we had to ensure that all cellular iron reserves in P. aeruginosa were depleted so that P. aeruginosa strictly utilizes the iron source supplied in the screening medium. To this end, P. aeruginosa strain PAO1 was first grown in LB broth for 4 h to mid-exponential phase (Fig. 1A), and cells from this phase were then inoculated at a high cell density into defined succinate morpholinepropanesulfonic acid (MOPS) medium (SMM) containing no iron source (SMM-N). After 3 h of growth in SMM-N, all internal iron reserves of P. aeruginosa had been depleted, as there was no change in the optical density (OD) (Fig. 1B). This regimen of iron depletion was strictly followed before performing all growth experiments.

**FIG 2:**
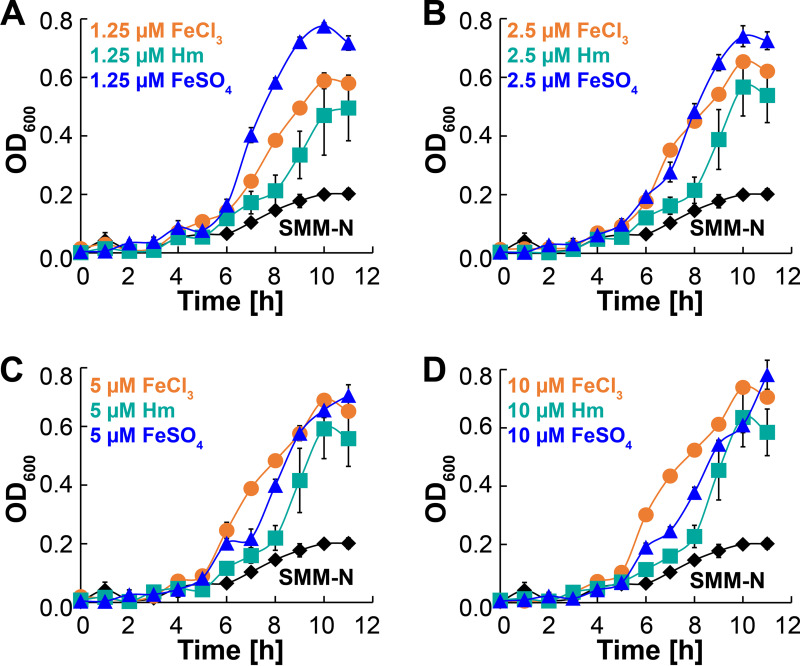
Iron-specific growth conditions of P. aeruginosa. Shown is the growth of iron-depleted PAO1 cells in SMM-N containing ferric chloride (FeCl_3_), hemin (Hm), or ferrous sulfate (FeSO_4_) at a final concentration of either 1.25 μM (A), 2.5 μM (B), 5 μM (C), or 10 μM (D). Iron-depleted PAO1 was also grown in just SMM-N (black diamonds) to determine background growth from any residual iron in the medium. Note that for all panels, data points for growth in SMM-N are the same. Error bars represent the SEM from three biological replicates. In many cases, the error bars are smaller than the marker data points.

To develop the HTS assay, we first determined the growth conditions for the HTS medium. These growth conditions needed to establish two parameters: (i) the concentration of FeCl_3_, Hm, or FeSO_4_ to be used in the screening medium where the growth kinetics of P. aeruginosa are comparable under all three conditions and (ii) the readout time when P. aeruginosa reaches maximum growth under all three growth conditions. Knowing these conditions would allow us to appropriately identify molecules that inhibit the growth of P. aeruginosa. The screening medium for the HTS assay was prepared by adding FeCl_3_, Hm, or FeSO_4_ to the base medium (SMM-N). We opted not to use artificial sputum medium (ASM) (26–33) for the screen, which is typically used in P. aeruginosa and CF research. Instead, we utilized a chemically defined medium (SMM-N) because this allows us to manipulate the iron source and concentration, which is not possible with ASM. Ascorbate (a reducing agent) was added to the FeSO_4_ medium, and plates were wrapped with parafilm to maintain FeSO_4_ in its reduced form, as done in previous studies for FeSO_4_ growth experiments (10). To determine the optimal HTS assay conditions, in 96-well plates, iron-depleted P. aeruginosa was inoculated into SMM-N containing various concentrations of FeCl_3_, Hm, or FeSO_4_, and growth was recorded by monitoring the OD at 600 nm (OD_600_) (Fig. 2). Since it is impossible to remove all traces of iron from the medium, iron-depleted P. aeruginosa cells were also inoculated into just SMM-N to determine the background growth from residual iron in the base medium. Compared to the background growth, we observed that P. aeruginosa reached maximal growth at 10 h regardless of the iron source or concentration (Fig. 2), which also demonstrated that ascorbate does not influence the growth kinetics of P. aeruginosa. However, P. aeruginosa achieved very similar maximal growth at 10 h with all iron sources at a final concentration of 5 μM (Fig. 2C). Based on these observations, we determined that in the HTS assay, the optimal concentration for all iron sources is 5 μM, and the optimal time for readout is 10 h.

### Determining the robustness of the high-throughput screening assay.

**FIG 3:**
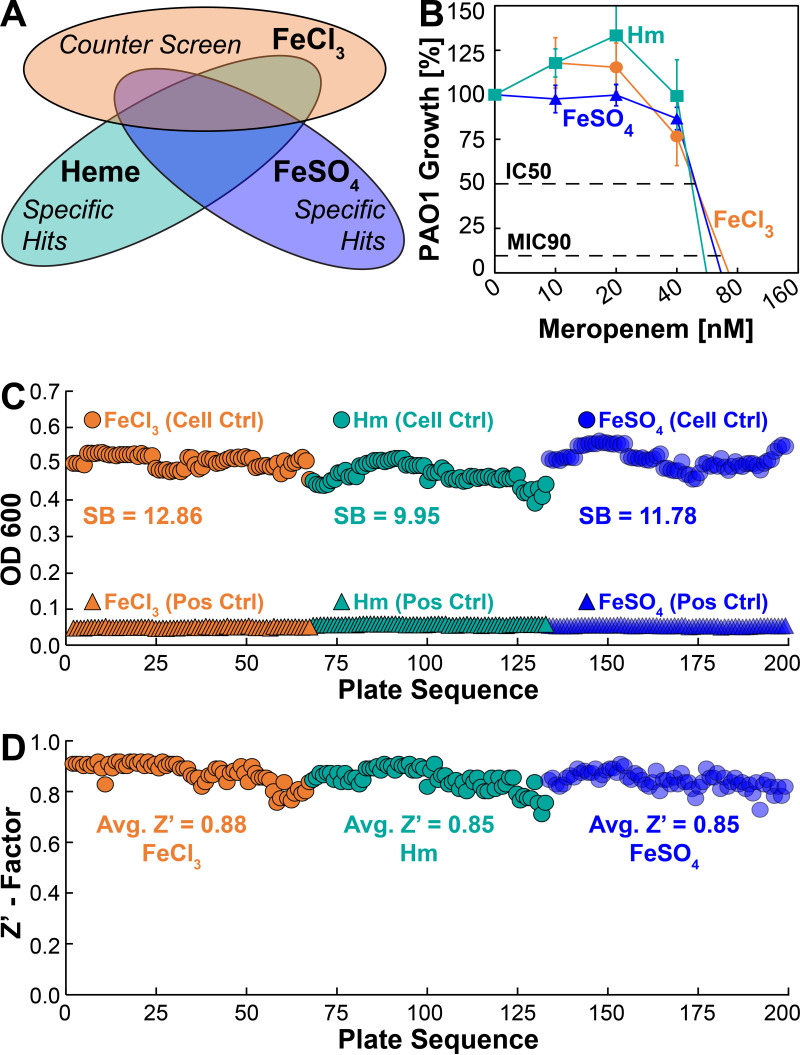
High-throughput screening (HTS) assay performance. (A) Schematic representation of the three growth conditions used for high-throughput screening of molecules. (B) Survival of PAO1 in SMM-N containing 5 μM FeCl_3_ (orange circles), 5 μM Hm (cyan squares), or 5 μM FeSO_4_ (blue triangles) in the presence of increasing concentrations of meropenem. PAO1 growth was monitored by measuring the endpoint OD_600_ at 10 h, and the percentage of growth was determined relative to the growth of PAO1 in the absence of meropenem. Error bars represent the SEM from five biological replicates. (C) Iron-depleted PAO1 was grown in 384-well plates for 10 h in SMM containing 5 μM FeCl_3_, 5 μM Hm, or 5 μM FeSO_4_ in the absence (cell control) or presence (positive control) of meropenem. Control samples were arranged in 384-well plates in opposing quadrants. SB, signal-to-background ratio. (D) The Z′-factor was calculated for all plates, and the average (Avg.) Z-factor was determined under all three growth conditions.

As stated above, our HTS approach involves screening a compound library simultaneously for antipseudomonal activity with three different iron sources (Fig. 3A). To verify the robustness of our assay conditions, we determined the maximum and minimum growth of P. aeruginosa in 384-well HTS plates. P. aeruginosa was inoculated into SMM-N containing 5 μM FeCl_3_, Hm, or FeSO_4_ and grown for 10 h to determine maximum growth. Since PAO1 is susceptible to meropenem under all growth conditions (Fig. 3B), we used 20 μM meropenem as a positive control for growth inhibition to determine minimum growth. We used the OD_600_ as a quantitative readout because it is inexpensive, requires few handling steps, and is less prone to manipulation-induced variations. Based on maximum and minimum growth, we observed a strong signal-to-background (SB) ratio of >9 under all growth conditions (Fig. 3C). The quality and performance of our HTS assay were determined by calculating the Z′-factor, which must ideally be in the range of 0.5 to 1.0 to be considered robust enough for HTS (34). Under all growth conditions, the Z′-factor for the 384-well format was >0.8 (Fig. 3D), demonstrating that our assay is highly reliable and reproducible.

### A targeted whole-cell high-throughput screening assay to identify inhibitors of heme and ferrous iron acquisition by P. aeruginosa.

We performed a small proof-of-concept screen using a library of structurally diverse small molecules that serve as a basis for new lead discovery in HTS and to identify new activities in established drugs. Our goal was to demonstrate that our targeted whole-cell screening approach can identify molecules that inhibit the Hm and ferrous iron acquisition systems of P. aeruginosa. The small-molecule library was simultaneously screened against P. aeruginosa under the three growth conditions, where the FeCl_3_ growth condition served as a counterscreen (Fig. 3A). In principle, each screen serves as a counterscreen for the others. Molecules that exhibit antipseudomonal activity in the presence of only Hm or FeSO_4_ are considered Hm-specific inhibitors (HSIs) or ferrous iron-specific inhibitors (FSIs), respectively (Fig. 3A). Using this counterscreening process of elimination, we identified three HSIs, one FSI, and two molecules that inhibit the growth of P. aeruginosa in both FeCl_3_ and Hm, which is a hit rate of ~0.3%.

**FIG 4:**
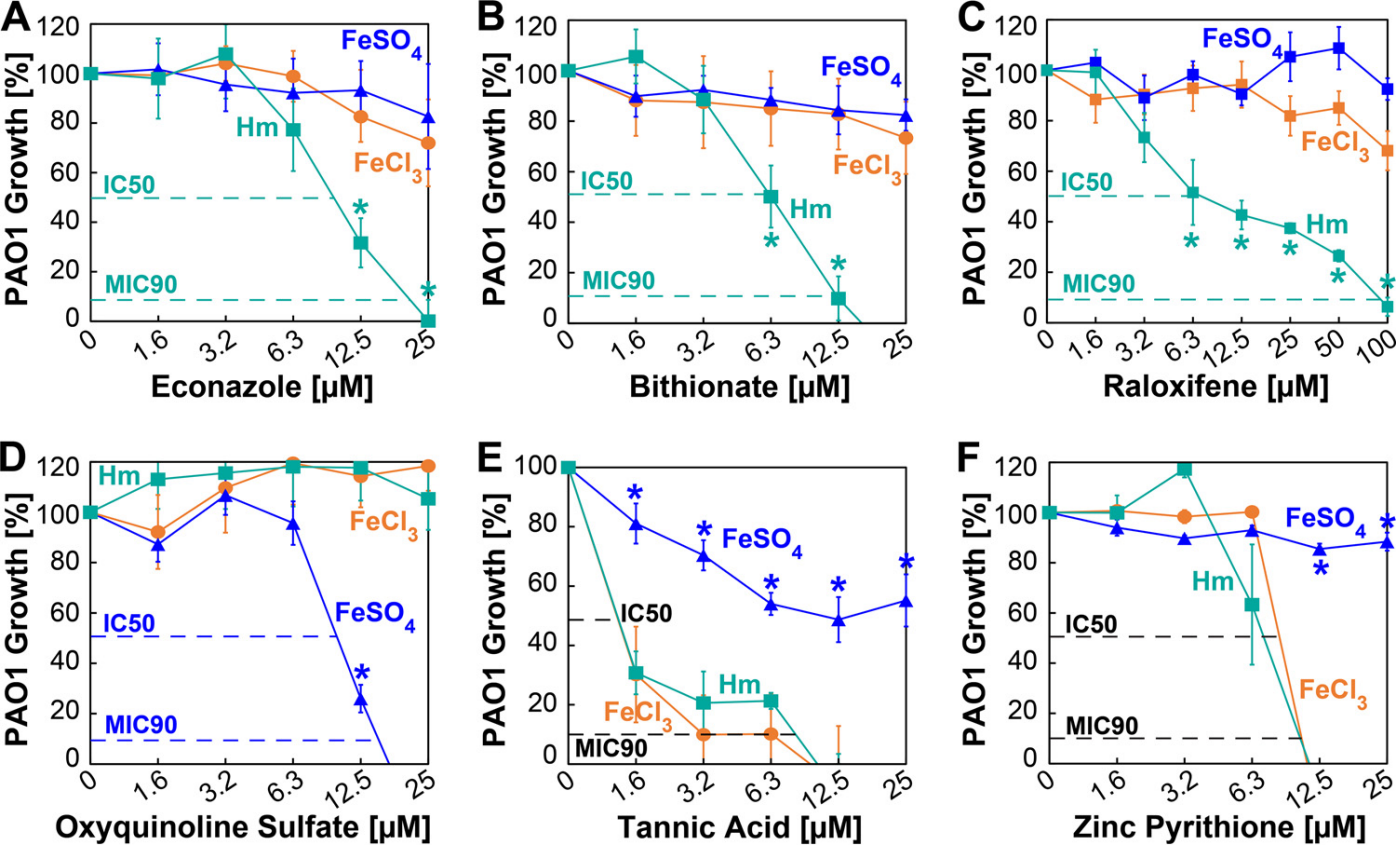
Specific activity of hit molecules determined by dose-response assays. Shown is the survival of PAO1 in SMM-N containing 5 μM FeCl_3_ (orange circles), 5 μM Hm (cyan squares), or 5 μM FeSO_4_ (blue triangles) in the presence of increasing concentrations of econazole (A), bithionate (B), raloxifene (C), oxyquinoline sulfate (D), tannic acid (E), and zinc pyrithione (F). PAO1 growth was monitored by measuring the endpoint OD_600_ at 10 h, and the percentage of growth was determined relative to the growth of PAO1 in the absence of any hit molecules. Error bars represent the SEM from five biological replicates. Growth data points with asterisks are significantly different from growth under the other iron conditions. Significance was determined by Tukey’s HSD test following an *F* test (*P* < 0.05).

We determined that econazole (Fig. 4A), bithionate (Fig. 4B), and raloxifene (Fig. 4C) specifically inhibit the growth of P. aeruginosa in the presence of only Hm as the sole iron source but do not exhibit any activity against P. aeruginosa in the presence of either FeCl_3_ or FeSO_4_. Dose-response curves indicated 50% inhibitory concentrations (IC_50_) of ~10 μM for econazole and 6.3 μM for bithionate and raloxifene and MIC_90_s of ~20 μM and 12.5 μM for econazole and bithionate, respectively. While the MIC_90_ for raloxifene in Hm was achieved only at a very high concentration of ~100 μM, it was still inactive in the presence of FeCl_3_ and FeSO_4_. We also determined that oxyquinoline sulfate (OS) (Fig. 4D) specifically inhibits the growth of P. aeruginosa only in the presence of FeSO_4_ as the sole iron source but does not exhibit any activity against P. aeruginosa in the presence of either FeCl_3_ or Hm. Dose-response curves indicated an IC_50_ of ~11 μM and an MIC_90_ of <20 μM for oxyquinoline sulfate. Surprisingly, we also identified two molecules that inhibit the growth of P. aeruginosa in the presence of both FeCl_3_ and Hm. Tannic acid (TA) inhibits the growth of P. aeruginosa in FeCl_3_ and Hm, with an IC_50_ of ~1 μM and an MIC_90_ of <10 μM (Fig. 4E). However, tannic acid also exhibits some antipseudomonal activity in the presence FeSO_4_ albeit to a much lesser extent, suggesting that it can also exert inhibitory activity against P. aeruginosa growth in an iron-independent manner. Zinc pyrithione (ZPT) exhibits very similar antipseudomonal activities in both FeCl_3_ and Hm, with an IC_50_ of ~10 μM and an MIC_90_ of <12 μM (Fig. 4F). Unlike tannic acid, zinc pyrithione does not exhibit any activity against P. aeruginosa in the presence of FeSO_4_. Altogether, these results demonstrate that our targeted whole-cell screening approach successfully identified molecules that inhibit specific iron acquisition mechanisms of Pseudomonas aeruginosa.

### Activity of heme- and ferrous iron-specific inhibitors.

**FIG 5:**
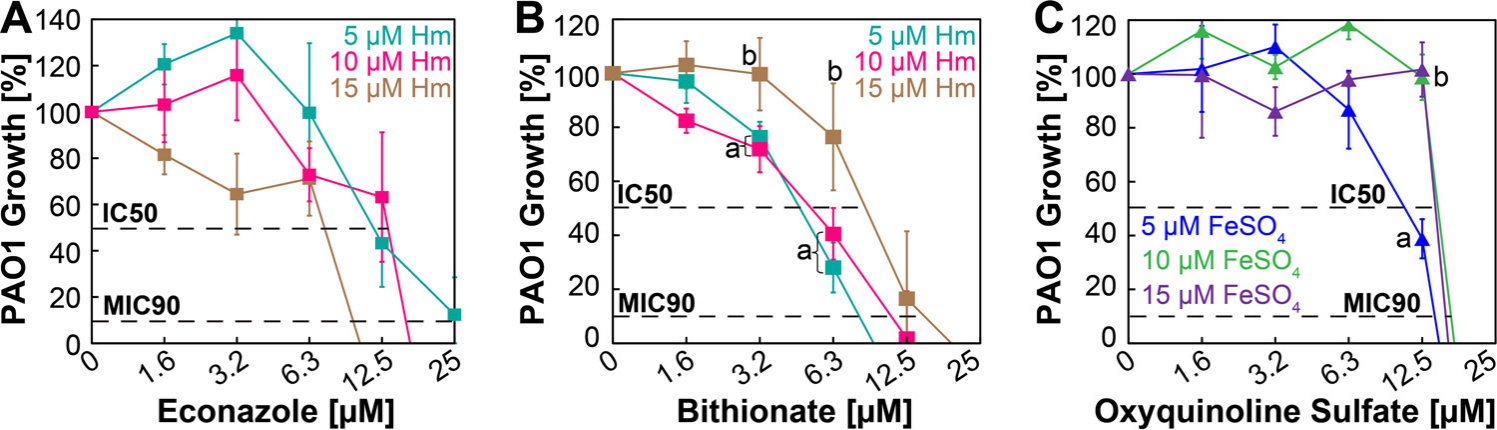
Effect of iron concentrations on heme- and FeSO_4_-specific inhibitors. (A and B) Survival of PAO1 in SMM-N containing 5 μM, 10 μM, or 15 μM Hm in the presence of increasing concentrations of econazole (A) or bithionate (B). (C) Survival of PAO1 in SMM containing 5 μM, 10 μM, or 15 μM FeSO_4_ in the presence of increasing concentrations of oxyquinoline sulfate. PAO1 growth was monitored by measuring the endpoint OD_600_ at 10 h, and the percentage of growth was determined relative to the growth of PAO1 in the absence of any hit molecules. Error bars represent the SEM from five biological replicates. Plots with different letters are significantly different as determined by Tukey’s HSD test following an *F* test (*P* < 0.05).

The utilization of Hm and ferrous iron invariably starts with their transport into the cell across the outer and inner membranes of P. aeruginosa by specific membrane transporter proteins (35). It is possible that these transporters are being blocked by HSI and FSI molecules preventing the uptake of Hm and FeSO_4_, respectively. Alternatively, it may be that the HSI and FSI molecules direct bind Hm or FeSO_4_, which prevents access of P. aeruginosa to the iron source. If the molecules are blocking transporter proteins, we hypothesized that increasing the iron levels would not recover the growth of P. aeruginosa. If the inhibitors are preventing access to Hm and FeSO_4_ through binding, we hypothesized that increasing the iron levels would then recover the growth of P. aeruginosa. To establish a possible mechanism, we determined the ability of the hit molecules to block P. aeruginosa growth in the presence of increasing concentrations of Hm or FeSO_4_. We analyzed only the hit molecules with MIC_90_ activity (econazole, bithionate, and oxyquinoline sulfate). The antipseudomonal activity of econazole remains the same at 5 μM and 10 μM Hm (IC_50_ ~12.5 μM), and its activity increases at 15 μM Hm (IC_50_ <12.5 μM) (Fig. 5A). Interestingly, the MIC_90_ for econazole decreases with increasing concentrations of Hm (Fig. 5A). The antipseudomonal activity of bithionate remains the same at 5 μM and 10 μM, with no statistically significant differences (Fig. 5B), but at 15 μM Hm, the antipseudomonal activity of bithionate is partially relieved, with the IC_50_ increasing to ~10 μM, which was statistically significant (Fig. 5B). These observations indicate that econazole and bithionate inhibit the growth of P. aeruginosa in an Hm-dependent manner but likely by different mechanisms. For the FSI oxyquinoline sulfate, there is only a marginal reduction (IC_50_ ~14 μM) in antipseudomonal activity after increasing the FeSO_4_ concentration to 10 μM and 15 μM (Fig. 5C). However, the MIC_90_ for oxyquinoline sulfate remains mostly unchanged (Fig. 5C), indicating that increasing the FeSO_4_ concentration only marginally alleviates the FeSO_4_-dependent antipseudomonal activity of oxyquinoline sulfate.

### Effects of HSI and FSI molecules on meropenem activity.

**FIG 6:**
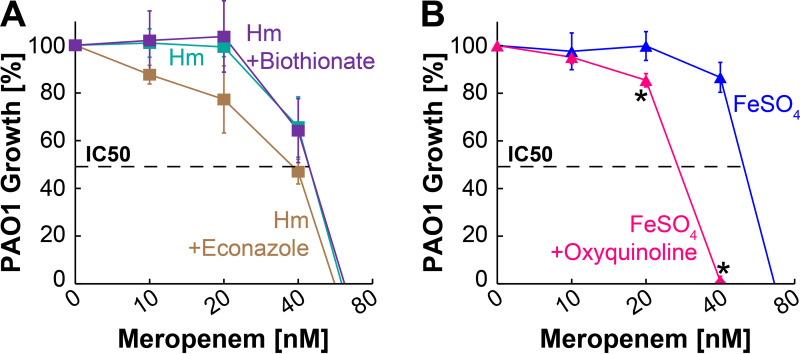
Effect of heme- and FeSO_4_-specific inhibitors on the activity of meropenem. (A) Survival of PAO1 in SMM-N containing 5 μM Hm (cyan), 5 μM Hm and 3 μM econazole (brown), or 5 μM Hm and 3 μM bithionate (purple) in the presence of increasing concentrations of meropenem. (B) Survival of PAO1 in SMM-N containing 5 μM FeSO_4_ (blue) or 5 μM FeSO_4_ and 3 μM oxyquinoline sulfate (pink) in the presence of increasing concentrations of meropenem. Asterisks denote significant differences as determined by Tukey’s HSD test following an *F* test (*P* < 0.05), compared to growth in just FeSO_4_. PAO1 growth was monitored by measuring the endpoint OD_600_ at 10 h, and the percentage of growth was determined relative to the growth of PAO1 in the absence of any meropenem. Error bars represent the SEM from five biological replicates. Note that data points for Hm and FeSO_4_ are the same as those in Fig. 3B.

A key aspect of antipseudomonal chemotherapy is ensuring that any lead molecules coming out of the drug discovery pipeline do not exhibit antagonistic effects on commercially used antibiotics. Since PAO1 is susceptible to meropenem in the nanomolar range (Fig. 3A) and functions by inhibiting cell wall synthesis, we wanted to explore the effects of HSI and FSI molecules on the activity of meropenem. To this end, the antipseudomonal activity of meropenem at various concentrations was determined by growing PAO1 in the presence of 5 μM Hm and HSIs or 5 μM FeSO_4_ and the FSI. All HSI and FSI molecules were used at a subinhibitory concentration (3 μM), which is still in molar excess compared to the nanomolar concentrations of meropenem (Fig. 6). In the presence of the HSI bithionate, the inhibitory activity of meropenem against P. aeruginosa was completely unchanged (Fig. 6A). In the presence of the HSI econazole, the inhibitory activity of meropenem was slightly potentiated, as the growth of P. aeruginosa was reduced at lower concentrations of meropenem (Fig. 6A). However, this difference was not statistically significant. Interestingly, addition of the FSI oxyquinoline sulfate significantly potentiated the inhibitory activity of meropenem against P. aeruginosa. The IC_50_ of meropenem is ~60 nM against P. aeruginosa when grown in FeSO_4_, but the addition of oxyquinoline sulfate potentiates the inhibitory activity of meropenem by ~2-fold and reduces the IC_50_ to ~30 nM (Fig. 6B). These results demonstrate that the iron-specific inhibitors identified from our HTS do not interfere with the activity of meropenem and that they can also potentiate the activity of meropenem.

## DISCUSSION

P. aeruginosa is strictly dependent on the acquisition of iron within the human host for survival and virulence because iron is an essential micronutrient required for vital biological processes (36). P. aeruginosa uses three redundant iron acquisition mechanisms to overcome iron limitation in the host: (i) siderophore-mediated iron acquisition (SMIA) to chelate ferric iron (Fe^3+^) from host Tf, Lf, and ferritin; (ii) Hm-mediated iron acquisition (HIA) to sequester host Hm iron; and (iii) ferrous iron-mediated iron acquisition (FIA) to directly transport ferrous iron (Fe^2+^). As stated above, major efforts have been made to identify inhibitors of P. aeruginosa SMIA, but research to identify inhibitors of HIA and FIA is lacking. In this study, we present a proof of concept that a targeted whole-cell screening approach can be employed to directly identify inhibitors of P. aeruginosa HIA and FIA. We utilized a novel targeted conditional screening approach to identify molecules that specifically target and inhibit HIA and FIA by P. aeruginosa. This approach relies on screening a small-molecule library for antipseudomonal activity by growing P. aeruginosa under different growth conditions where the exogenously added iron source is the only variable in the medium. Each growth condition serves as a counterscreen for the other to specifically identify either HIA or FIA inhibitors. We have termed these inhibitors Hm-specific inhibitors (HSIs) and ferrous iron-specific inhibitors (FSIs), respectively. Utilizing this new approach, we identified three HSIs (econazole, bithionate, and raloxifene) that exhibit antipseudomonal activity only in the presence of Hm iron and one FSI (oxyquinoline sulfate [OS]) that exhibits antipseudomonal activity only in the presence of ferrous iron (Fig. 4). The iron-specific activity of these molecules is further supported by the observation that these molecules do not exhibit any antipseudomonal activity under the counterscreening conditions.

We also attempted to determine the possible mechanism of action of the most active HSIs and FSI. Our results show that increasing the iron concentration in the medium only marginally reduced or did not affect the antipseudomonal activity of these molecules (Fig. 5). This suggests that these inhibitors likely do not prevent access to the iron source through directly binding Hm and ferrous iron, but this possibility cannot be discounted. It is well known that the primary mode of action of azole drugs such as econazole is mediated by binding the Hm cofactor (37) to inactivate cytochrome P450 enzymes, which are essential for diverse catalytic roles (38). Since P. aeruginosa has genes that encode P450 enzymes (39) and because it has been shown that econazole can bind these P. aeruginosa P450 enzymes (40), it is possible that econazole could inactivate the P. aeruginosa P450 enzymes and inhibit growth. If this was the case, we would expect to also observe the antipseudomonal activity of econazole in the presence of FeCl_3_ or FeSO_4_ and not just specifically in the presence of Hm (Fig. 4A). In P. aeruginosa, outer membrane receptors first bind Hm and transport it into the periplasm, where Hm is bound by a substrate binding protein and then transported across the inner membrane by a dedicated ABC transporter (12). It is conceivable that econazole could bind to the protein-Hm complex at any point in this transport process and prevent Hm entry into the cytoplasm. Since increasing the Hm concentration did not alleviate the inhibitory activity of econazole, we speculate that econazole likely blocks the transport of Hm into the cytoplasm. The second HSI, bithionate, has higher antipseudomonal activity (IC_50_ ~6 μM; MIC_90_ ~12 μM) than econazole. Since increasing the Hm concentration to 15 μM in medium partially alleviated some of the antipseudomonal activity of bithionate, it may be that bithionate binds Hm and prevents access to Hm. We also identified raloxifene as another highly specific HSI but with much lower activity than econazole and bithionate. Interestingly, a previous study by Ho Sui et al. identified raloxifene as an antipseudomonal that prevents the production of pyocyanin (41), which is a siderophore required for ferric iron acquisition (12, 35). The growth and infection assays in the study by Ho Sui et al. were performed with 100 μM raloxifene, which is the same concentration where we observe ~30% inhibition in the presence of ferric chloride in our study (Fig. 4C). However, our specific growth conditions clearly show that raloxifene has much higher Hm-specific activity. To the best of our knowledge, our study is also the first to directly attempt to identify ferrous iron acquisition inhibitors, and we show that OS specifically inhibits the growth of P. aeruginosa in the presence of FeSO_4_. OS and its derivatives are known antibacterial agents that can bind different metals (42–45) and have been shown to exhibit increased antibacterial activity in the presence of ferrous iron salts (46). Based on these observations, it is likely that the antipseudomonal activity of OS is partially due to the sequestration of ferrous iron because increasing the FeSO_4_ concentration from 5 μM to 10 μM allowed P. aeruginosa to grow at 12.5 μM OS (Fig. 5C). On the same note, increasing the FeSO_4_ concentration from 10 μM to 15 μM did not allow P. aeruginosa to survive significantly better. In fact, for all FeSO_4_ concentrations, the MIC_90_ of OS was only marginally affected (Fig. 5C), suggesting that OS may also inhibit growth by other mechanisms.

An unexpected finding of our HTS assay was the observation that tannic acid (TA) and zinc pyrithione (ZPT) inhibited the growth of P. aeruginosa in the presence of both FeCl_3_ and Hm. Previous studies have shown that these molecules can be potent inhibitors of P. aeruginosa during both planktonic and biofilm growth (47, 48). Since TA and ZPT are known metal-chelating compounds (49, 50), it is likely that they inhibit P. aeruginosa growth in FeCl_3_ through ferric iron chelation. TA also forms ferrous iron complexes with a lower affinity at neutral-to-alkaline pH. Our buffered (pH 6.8) growth medium may allow some Fe^2+^-TA complexation, which could explain the partial growth defect observed under the FeSO_4_ conditions (Fig. 4E). A more confounding observation is how TA and ZPT inhibit the growth of P. aeruginosa in the presence of Hm. It is not known whether TA can bind and sequester Hm, and it is unlikely that TA can chelate iron from the porphyrin ring because porphyrin tightly binds iron. The only known mechanism of iron release from Hm is through the cleavage of the porphyrin ring by dedicated heme monooxygenase enzymes (51). On the other hand, there is a plausible explanation for ZPT-mediated Hm inhibition. ZPT is a commonly used antifungal, and previous studies have shown that it inhibits yeast growth by inactivating iron-sulfur (Fe-S) proteins (52). Since Fe-S cluster biogenesis is intimately linked to heme biogenesis and metabolism (53), this further leads to mitochondrial dysregulation and growth inhibition (54). It is possible that ZPT may similarly impact Fe-S proteins and heme metabolism in P. aeruginosa. However, Fe-S proteins perform vital cellular functions under all conditions, further raising the question of why the ZPT-mediated inhibition of Fe-S proteins would not inhibit P. aeruginosa growth in the presence of FeSO_4_. An alternative explanation could be that TA and ZPT inhibit some component(s) that is shared between the ferric and heme iron acquisition pathways, which in this case would be the TonB-ExbBD proteins. Transport of the Fe^3+^-siderophore complex or Hm across the bacterial outer membrane is an active process requiring energy, which is provided by the TonB-ExbBD proteins (51). Therefore, it is tempting to speculate that TA and ZPT could be inhibiting TonB-ExbBD function in some manner. Ferrous iron is transported by porins by passive diffusion in a TonB-independent manner, which could explain the significantly reduced activity of TA and the inactivity of ZPT in the presence of FeSO_4_.

Altogether, the results of our study show that our targeted whole-cell HTS approach identifies molecules that block specific iron acquisition systems and that these iron-specific inhibitors are structurally diverse (see Fig. S1 in the supplemental material), and we can identify new roles for previously characterized iron inhibitors. There are some limitations to the current study. We recognize that one limitation is that the molecules identified from this screen exhibit micromolar levels of antipseudomonal activity. We do not see this as a disadvantage because our primary goal was to show a proof of concept for our targeted whole-cell approach, and we successfully proved that our approach is logical and performs robustly. We realize that antifungals such as econazole are cytotoxic and would not be used for actual chemotherapy. However, from this pilot screen, we were able to identify structurally diverse molecules that can serve as a platform for the rational design or synthesis of more targeted compound libraries for future high-throughput screens. Another limitation of our study is that our screening was performed under very specific growth conditions. A reasonable argument could be made that the screening should have been performed in artificial sputum medium (ASM), which simulates the CF lung environment. To the best of our knowledge, there are at least eight different ASM (26–33) that are used in CF research, and the main drawback is that their iron source and levels cannot be manipulated. Our screening approach relies on the exogenous addition of iron sources (FeCl_3_, Hm, or FeSO_4_) to identify specific iron acquisition inhibitors. While Chelex treatment can remove free iron from the ASM, it does not remove iron complexed in proteins, and there is also a risk of chelating other metals. For these reasons, we opted to use a defined medium so that we could target specific iron acquisition systems. Furthermore, our screening was performed using the P. aeruginosa PAO1 isolate, and it is possible that the small molecules identified in our screen could exhibit various effects on other P. aeruginosa isolates. We also recognize that our screening was performed with planktonically grown P. aeruginosa, and it is well established that P. aeruginosa exists within biofilms, where its metabolism is very different. An advantage of our approach is that it is highly adaptable to any growth condition, and molecule screening can easily be performed against P. aeruginosa grown within biofilms. While it was not within the scope of this study, we fully intend to use this screen to identify iron-specific inhibitors of P. aeruginosa biofilms in future experiments.

A seminal study by Nguyen et al. showed that P. aeruginosa mutants defective in siderophore production successfully colonize the CF lung and that P. aeruginosa reduces siderophore production and upregulates the genetic components of heme and ferrous iron acquisition in the CF lung (11). These observations suggest that P. aeruginosa preferentially uses heme and ferrous iron over ferric iron within the CF lung environment. Blocking iron acquisition systems simultaneously *in vivo* would starve P. aeruginosa of an essential nutrient, which could increase the activity of other antipseudomonal antibiotics, as we have shown is the case with OS (Fig. 6D). Our whole-cell screening approach is advantageous because it directly identifies biologically active molecules that target heme and ferrous iron acquisition systems and overcomes the need for the *in silico* design of molecules based on structural analysis. Moreover, our screening approach to identify iron acquisition inhibitors is not limited to just P. aeruginosa and can be easily extended to any pathogen. Since Gram-negative pathogens share many similarities in iron acquisition at both the mechanistic and molecular levels (15, 22, 51, 55–58), our screening approach presents a significant opportunity to develop novel broad-spectrum iron acquisition inhibitors of Gram-negative pathogens, which pose an imminent global threat to human health (9, 59, 60). In conclusion, we envision the identification of highly active molecules that can be developed into a cocktail of iron acquisition inhibitors to block SMIA, HIA, and FIA not only in Pseudomonas aeruginosa but also in other Gram-negative pathogens.

## MATERIALS AND METHODS

### Bacterial strains, growth media, and molecules.

Wild-type Pseudomonas aeruginosa strain PAO1 (PAO1) was first routinely streaked onto solid LB agar plates and grown statically at 37°C for 24 h. Liquid cultures were then initiated from a single isolated colony in LB broth and grown with shaking at 200 rpm at 37°C. For growth assays, PAO1 was first iron depleted by growth in defined succinate MOPS medium (SMM) (61) containing no iron (SMM-N) and then inoculated into SMM-N containing specific iron sources. Iron-free SMM-N was prepared in 1 L of ultrapure Millipore water using the following reagents: 0.01 g of EDTA, 0.6 g of KH_2_PO_4_, 0.9 g of K_2_HPO_4_, 1.0 g of NH_4_Cl, 0.2 g of MgSO_4_ · 7H_2_O, 0.075 g of CaCl_2_ · 6H_2_O, 2.2 g of sodium succinate, 0.1 g of yeast extract, 0.4 g of glucose, 2.0 mL of trace elements, 2.0 mL of a vitamin solution, and 40 mM MOPS (pH 6.8). The trace elements solution was prepared in 1 L of Millipore water using the following reagents: 0.005 g of ZnSO_4_ · 7H_2_O, 0.003 g of MnCl_2_ · 4H_2_O, 0.002 g of H_3_BO_3_, 0.005 g of CoCl_2_ · 6H_2_O, 0.001 g of CuCl_2_ · 2H_2_O, 0.002 g of NiCl_2_ · 6H_2_O, and 0.003 g of Na_2_MoO_4_ · 2H_2_O. The vitamin solution was prepared in 1 L of Millipore water using the following reagents: 80 mg of biotin, 400 mg of thiamine-HCl · 2H_2_O, 400 mg of nicotinic acid, and 20 mg of vitamin B_12_. Reagents were always sterilized using a 0.2-μm filter and never autoclaved. Econazole (catalog number E09575G), bithionate (catalog number T086525G), raloxifene (catalog number R01091G), tannic acid (catalog number AA3641022), and zinc pyrithione (catalog number 50-199-8689) were purchased from Fisher Scientific. Oxyquinoline sulfate (catalog number 55100-100G-F) was purchased from Sigma.

### Iron depletion of P. aeruginosa and determination of growth conditions.

A liquid starter culture of PAO1 grown overnight was initiated by inoculating a single colony into 5 mL of LB broth. From the starter culture, PAO1 was then inoculated into 10 mL of fresh LB broth at an OD_600_ of 0.05. PAO1 cells were harvested from the mid-logarithmic phase (Fig. 1A), washed twice with 10 mL of phosphate-buffered saline (PBS), and then inoculated into 10 mL of SMM-N at an initial OD_600_ of 0.5. To deplete internal iron reserves, cells were allowed to grow in SMM-N until there was no change in the optical density (Fig. 1B). Iron-depleted PAO1 cells were harvested and washed twice with 10 mL of PBS. PAO1 cells were then inoculated into 96-well plates at an OD_600_ of 0.01 to a final volume of 200 μL in SMM containing various concentrations (Fig. 2) of ferric chloride (FeCl_3_), ferrous sulfate (FeSO_4_), or hemin as the sole iron source. For medium containing FeSO_4_, ascorbate (a reducing agent) was added to a final concentration of 2 mM, and plates were wrapped with parafilm to maintain FeSO_4_ in its reduced form, as done in previous studies (10). All plates were incubated at 37°C with shaking, and the optical density was monitored using a BioTek Synergy plate reader at 1-h intervals.

### High-throughput screening assay, compound library, and identification of hit molecules.

High-throughput screening (HTS) was performed at Southern Research (SR). A collection of 20,479 compound samples from Enamine and Microsource (Pharmakon 1600 library) was screened at a concentration of 10 μg/mL, 20 μg/mL, or 50 μM, with a dimethyl sulfoxide (DMSO) concentration of 0.4%. Compounds and the DMSO carrier were dispensed into 384-well plates in a 5-μL volume using the Biomek FX liquid handler (Beckman Coulter). Cells containing 0.4% DMSO served as the cell controls (Cell Ctrl), and cells containing 20 μg/mL meropenem served as the positive controls (Pos Ctrl). PAO1 was first iron depleted as mentioned above and then added to three identically dosed 384-well plates at an OD_600_ of 0.001 to a final volume of 35 μL in SMM containing either 5 μM FeCl_3_, 5 μM FeSO_4_, or 5 μM hemin, where the only variable among the three plates was the iron source. Plates were incubated at 37°C in a humidified atmosphere, and the OD_600_ was determined at 10 h using a CLARIOstar plate reader (BMG Labtech). Percent inhibition was calculated as 100 × {[compound OD_600_ value − median of the cell control]/[median of the positive (meropenem) control − median of the cell control]}. The single-concentration hit data were then analyzed to select molecules for dose-response assays at Oklahoma State University. Molecules that inhibited the growth of PAO1 by at least 50% under only hemin or FeSO_4_ growth conditions and were inactive under FeCl_3_ conditions were analyzed in dose-response assays.

### Determination of inhibition by hit molecules against P. aeruginosa using dose-response assays.

PAO1 was first grown and then iron depleted as mentioned above. Washed PAO1 cells were inoculated into 96-well plates at an OD_600_ of 0.001 to a final volume of 200 μL in SMM containing 5 μM FeCl_3_, 5 μM FeSO_4_, or 5 μM hemin as the sole iron source and increasing concentrations of hit molecules. All plates were incubated at 37°C with shaking, and the optical density was determined at 10 h using a BioTek Synergy plate reader.

### Statistical analysis.

SigmaPlot (Systat Software) was used for statistical analysis. Where applicable, statistical significance was determined by Tukey’s honestly significant difference (HSD) test following an *F* test. *P* values of less than 0.05 are considered significant. All data presented are mean values, with error bars representing standard errors of the means (SEM), from at least three biological replicates.

### Data availability.

All source data files are provided and are also available to anyone upon request. All requests should be addressed to Avishek Mitra.
