# Supplementary material for: A Novel Approach To Identify Inhibitors of Iron Acquisition Systems of Pseudomonas aeruginosa
Source: Microbiol Spectr. 2022 Sep 13;10(5):e02437-22. doi: 10.1128/spectrum.02437-22 (PMC9604216; doi:10.1128/spectrum.02437-22)
Supplement: Supplemental file 1 — Fig. S1. Download spectrum.02437-22-s0001.pdf, PDF file, 0.1 MB [file spectrum.02437-22-s0001.pdf]

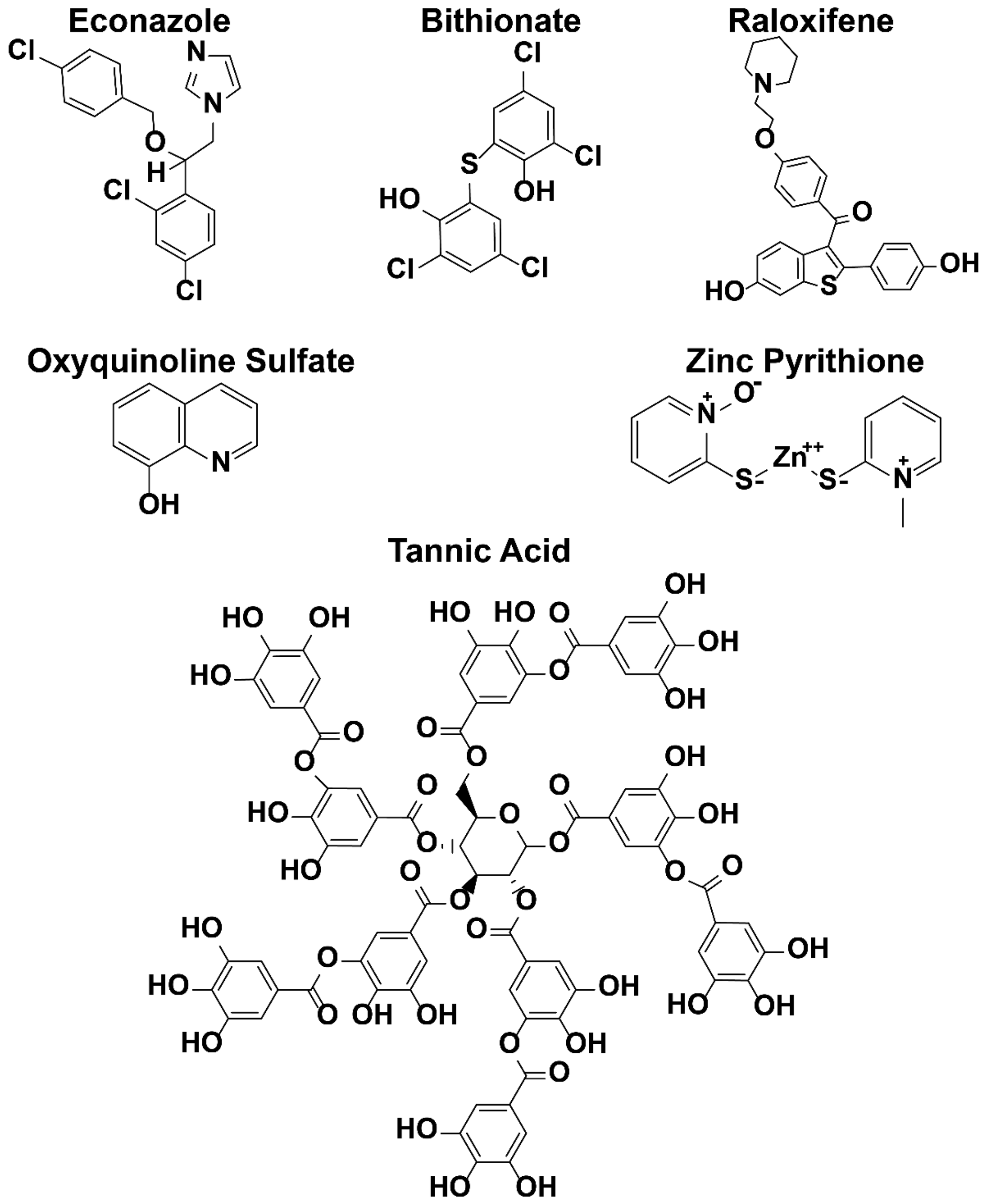

Figure S1. Structures of iron-specific inhibitors identified from targeted whole cell high throughput screening assay. Econazole, bithionate and raloxifene are heme-specific inhibitors of *Pa*. Oxyquinoline sulfate is a ferrous-specific inhibitor of *Pa*. Zinc pyrithione and tannic acid inhibit growth of *Pa* in the presence of both ferric and heme iron.
